# Safety and feasibility study of ex vivo expanded allogeneic-NK cells infusion in patients with acute pneumonia caused by COVID-19

**DOI:** 10.1186/s40814-023-01355-6

**Published:** 2023-08-04

**Authors:** Mohammad Ahmadvand, Mahdieh Shokrollahi Barough, Zahra Sadat Hashemi, Hassan Sanati, Fereshteh Abbasvandi, Masud Yunesian, Keivan Majidzadeh-A, Jalil Makarem, Hamid Reza Aghayan, Atefeh Abedini, Ardeshir Ghavamzadeh, Ramin Sarrami Forooshani

**Affiliations:** 1 ATMP Department, Breast Cancer Research Center, Motamed Cancer Institute, ACECR, Tehran, Iran; 2 Hematology, Oncology and Stem Cell Transplantation Research Center, Tehran, Iran; 3 Department of Environmental Health Engineering, School of Public Health, Tehran University of Medical Sciences, Tehran, Iran; 4 Genetics Department, Breast Cancer Research Center, Motamed Cancer Institute, ACECR, Tehran, Iran; 5 Department of Anesthesiology and Critical Care, Imam Khomeini Hospital Complex, Tehran University of Medical Sciences, Tehran, Iran; 6 Cell Therapy and Regenerative Medicine Research Center, Endocrinology and Metabolism Molecular-Cellular Sciences Institute, Tehran University of Medical Sciences, Tehran, Iran; 7 Chronic Respiratory Diseases Research Center, National Research Institute of Tuberculosis and Lung Diseases, Masih Daneshvari Hospital, Shahid Beheshti University of Medical Sciences, Tehran, Iran

**Keywords:** NK cells, COVID-19, Pneumonia

## Abstract

**Background:**

NK cells are the most active innate immune cells in antiviral immunity, which are impaired by SARS-COV2 infection. Infusion of allogeneic NK cells might be a complementary treatment to boost immune system function in COVID-19 patients. In this project, we focused on COVID-19 patients with low inspiratory capacity (LIC). This project aims to evaluate the feasibility and safety of allogeneic NK cell infusion as an intervention for respiratory viral disease.

**Methods:**

A non-blind two arms pilot study was designed and conducted after signing the consent form. Ten matched patients, in terms of vital signs and clinical features, were enrolled in the control and intervention groups. Approximately 2 × 10^6 cells/kg of NK cells were prepared under GCP (good clinical practice) conditions for each patient in the intervention group. The control group was under the same conditions and drug regimen except for the treatment with the prepared cells. Then, infused intravenously during 20 min in the ICU ward of Masih Daneshvari Hospital. The clinical signs, serological parameters, and CTCAE (Common Terminology Criteria for Adverse Events) were recorded for safety evaluation and the feasibility of project management were evaluated via designed checklist based on CONSORT.

**Results:**

There were no symptoms of anaphylaxis, hypersensitivity, significant changes in blood pressure, cardiovascular complications, and fever from injection time up to 48 h after cell infusion. The mean hospitalization period in the control and intervention groups was 10 and 8 days, respectively. The blood O2 saturation level was raised after cell infusion, and a significantly lower mean level of inflammatory enzymes was observed in the intervention group following discharge compared to the control group (*p* < 0.05). The inflammatory parameters differences at the discharge date in cell therapy group were highly negative.

**Conclusion:**

Intravenous infusion of ex vivo-expanded allogeneic NK cells was safe and feasible. However, the efficacy of this approach to reducing the severity of disease in COVID-19 patients with LIC could not be determined.

**Trial registration:**

Name of the registry: NKCTC. IRCT20200621047859N2. December 29, 2020. URL of trial registry record: https://www.irct.ir/trial/49382

**Supplementary Information:**

The online version contains supplementary material available at 10.1186/s40814-023-01355-6.

## Key messages regarding feasibility

- This project is focused on safety evaluation based on CTCAE and feasibility of the allogeneic NK cell infusion in acute respiratory disease caused by SARS-COV2 infectious using patients related information accessibility during the study and cell production facilities.
- Efficacy of NK cell therapy would be elucidated after feasibility and safety assessments, conducting a study with a larger population.
- The most expected implication of the present study was the good feasibility of the allogeneic NK cell transplantation in viral disease and lack of any complication.

## Introduction

Natural killer (NK) cells employ several mechanisms to kill the virus-infected cells. Natural killer (NK) cells are the most effective lymphocytes, for antiviral responses of the innate immune system and employ several mechanisms to eliminate the virus-infected cells. Apoptosis is induced via exocytosis by the cytotoxic granules, containing perforin and granzymes and some signaling pathways which are mediated by death receptor-ligand such as FAS-FASL and TRAIL interactions expressed on target cells and NK cells [1–3]. The anti-SARS-COV2 responses of NK cells may be observed due to the presence of NCR family (NKp46, NKp44, and NKp30) receptors [4], activation of other receptors such as CD57, or upregulation of NKg2C. NK cells could also contribute to the antiviral responses by releasing a wide range of antiviral pro-inflammatory cytokines such as interferon-alpha and gamma (IFN-α, IFN-γ) and activation in the Th1 immune response. These cytokines can enhance the activation of viral-specific cytotoxic T lymphocytes (CTL) [2, 5]. Some viral infections, such as cytomegalovirus (CMV) and encephalomyocarditis virus (EMCV), can trigger the expression of death receptors on infected cells [6, 7]. NK cells are greatly reduced in the peripheral blood of patients with viral infections, and patients with NK cell deficiency are more likely to become infected [8, 9]. While NK cell deficiency in viral infection is a rare condition, COVID-19 has been reported in several cases with deficient NK cells. It has been demonstrated that the pathogenic source of the disease could lead to decreased NK population and enhanced susceptibility to the virus; The NK cell infusion in COVID-19 is not only for viral load decrement, it can boost the adaptive immune system arm against infectious exacerbation [10, 11]. Advanced therapies such as immune cell therapy can boost the NK cell deficiency in viral infections and it can progress the healing of patients. Nowadays, the intravenous infusion of ex vivo-expanded NK cells is performed by either autologous or allogeneic sources of peripheral blood mononuclear cells (PBMCs) or cord blood [9, 10, 12]. A recent clinical trial about NK cell therapy for COVID-19 patients (NCT04578210) indicates that memorial NK cells from donors with a positive history of COVID-19 might be more effective than those from healthy donors [13]. The literature review of COVID-19 related cell therapy studies shows that focusing on reducing inflammation using mesenchymal stem cells in the lung is more important than eliminating the viral infection. The possibility of increased inflammation by natural killer cells might enhance the risk of NK cell infusion, so the administration of this treatment in patients with acute respiratory distress syndrome resulting from SARS-COV2 infectious COVID has been ruled out, and it is suggested only in patients with severe respiratory infection due to virus infection [9, 14]. Clinical trials approved for prescribing natural killer cells intravenous infusion in treating acute COVID-19 infections are negligible. The use of an allogeneic source in the preparation of natural killer cells for viral infectious diseases is very rare, and the effect of memory cells was studied only in the study of Lara Herrera et al., on a limited number of patients [13]. More clinical evidence is necessary to confirm that the injection of allogeneic natural killer cells is safe in respiratory viral infections, in order to be able to confirm the safety of these cells by not increasing the immunopathogenesis of the virus and then focus on its effectiveness. Korea’s GC Lab Company produced natural killer cells from umbilical cord blood for the first time, which they injected intravenously into patients with acute COVID-19 infection. Their study progressed to the second phase of the clinical trial and then introduced a product named CYNK001 COVID as an effective immune cell therapy for the treatment of COVID-19 [14, 15]. The high cost of production and the lack of receipt of cells by patients with acute respiratory distress syndrome prevented the development of this therapeutic product, but it opened the way for allogeneic cell therapies in COVID-19 treatments. It is imperative that the results of the safety and feasibility pilot study for these cells are judged before any conclusions about the effectiveness of cell therapy using natural killer cells. Thus, researchers in other fields can use these evidences to plan similar studies. The reason for focusing on the feasibility evaluation of project implementation in pilot studies is to be confident about the reproducibility of the results and to reach a good and appropriate sample size. This item requires innovation and planning according to the situation, which can be adapted from existing guidelines like CONSORT (defined in 2010) [16, 17]. In this study, we reported the results of the allogeneic NK cell infusion safety data in 5 patients with acute SARS-COV2 infectious and the vital sign and safety of an interventional study were assessed based on CTCAE (Common Terminology Criteria for Adverse Events) criteria following the NK cells infusion. NK cells were expanded from peripheral blood mononuclear cells of allogeneic healthy donors. This study is designed to assess the safety of NK cell infusion in patients with moderate respiratory distress. Since the patients with COVID-19-induced mediated respiratory distress do not respond to routine treatment protocols [18–21], combinatorial regimens like NK cell immuno-therapy might be more effective in the reduction of the disease symptoms and augmentation of the recovery rate. NK cells therapy could be a promising strategy for immune cell therapy of COVID-19.

## Materials and methods

### Aim, design, and setting of study

The main goal of this project was to evaluate the feasibility and safety of the allogeneic natural killer cell infusion in patients with an acute viral-associated respiratory infectious disease (VARID). The most important outcome we expect in this project is the confirmation of safety and feasibility for cell preparation and clinical trial execution. The safety of NK cell infusion was assessed by the CTCAE checklist and the feasibility of this intervention was considered as some parameters related to patient selection, sample size, and CONSORT-related criteria.

In this study, we conducted two arms of ICU-added COVID-19 patients with a low inspiratory capacity based on inclusion criteria. A CTCAE checklist was used to evaluate the vital signs and clinical parameters of the NK cell recipients. According to CTCAE, daily inflammatory signs such as fever, rash, heart function, respiratory capacity variations, and anaphylactic shock were closely monitored from the beginning of the infusion until 48 h after cell infusion. The design of this trial was reviewed by ethics committee referees and was licensed for 5 patients to evaluate the safety, feasibility, and clinical manifestations. This study was designed as a non-blinded two-arm clinical study. The control and intervention groups were matched in the case of the same clinical manifestation and critical criteria such as age, weight, O2 saturation level, and dependence on artificial respiration. This study was executed for two months in Masih Daneshvari Hospital (2020). The patient enrolment process and clinical trial steps were performed in the Intensive Care Unit (ICU) ward of Masih Daneshvari Hospital under the supervision of the Medical Ethics Committee and after obtaining the code of ethics (IR.ACECR.IBCRC.REC.1399.005). The study was also registered in the Iranian Registry of Clinical Trials as IRCT20200621047859N2. All patients have been familiarized with the project’s details and signed a written consent form before starting the cell infusion process. The Ethics committee manager checked the results of the trial and the treatment guideline would be updated for the patients in the phase II study.

### The criteria for feasibility evaluation

According to CONSORT guidelines, the following parameters were assessed for feasibility evaluation:

- The feasibility of cell production were evaluated for biological parameters such as Cell viability before cell infusion, immuno-phenotyping of final product in case of CD3 and CD56 population, and microbial quality controls for negative bacterial and fungal contamination and negative mycoplasma results
- The number of eligible patients, who are available to replace the excluded cases, is one of the criteria to ensure the feasibility of the sample size. This assessment was based on the rate of entry and exclusion of patients who were eligible according to the inclusion criteria. For this evaluation, the categorical scoring was allocated according to the entered and excluded number of patients into special care units; 1, every 2 days admission; 2, every day admission; 3, every day two patients admission.
- Following the NK cell infusion, accessibility of patient-related data was graded 1–5; 1, demographic information of a patient; 2, the patient’s hardcopy file; 3, Local access to the patient’s digital file; 4, web-based full access to the patient's information for long-term follow-up; 5, full access to patient information.
- Death cause evaluation access was graded from 0 to 2 for the deceased patients: 0, no access to cause of death; 1, general information of death causes that are included in the patient's file; 2, professional death causes declared by the evaluation of medical doctorate, which was associated with the trial. The patients-related samples were assessed for more clinical symptoms in both control and case groups. All assessments were obtained using a filled questionnaire by the main liaison researcher. Ethics Committee has considered this questionnaire (Supplementary Table S1).

### Patients

Ten patients with severe SARS-COV2 infection (confirmed by RT-PCR) were enrolled in this trial according to the inclusion criteria and divided to control and intervention groups. Patients were included based on the following criteria: male or female, aged 18–70 years old, approved pneumonia by chest imaging (ground-glass opacity in HRCT), laboratory confirmation of viral infection by reverse-transcription polymerase chain reaction (RT-PCR) from any diagnostic sampling source, normal transthoracic echocardiogram, PO2 /FIO2 = 100–300, with low inspiratory capacity/total lung capacity (LIC/TLC) ratio, and adequate kidney function defined by estimated glomerular filtration rate (CrCl) > 60 ml/min or ml/min/1.73 m^2^. Patients who have acquired the following criteria were excluded from the study: pregnancy or breastfeeding initiation, a positive record for HBV and, or HIV, a new systemic disease such as malignancy, or organ failure.

### Donor selection and blood sampling

Two healthy men volunteered for this study at the ages of 33 and 40 with O + and B + blood types, respectively. Active infection of SARS-COV2 in donors was evaluated after performing the gold standard multiplex real-time PCR test. Due to the negative results of this examination, active infection in these individuals was ruled out. The HIV, HBV, HCV, and blood culture results of donor samples were also negative. Finally, 70 ml of whole blood was obtained from donors by intravenous blood sampling using Venoject® blood collection syringes (Venoject-Terumo-Canada). Collected samples were transferred into heparinized falcon tubes (30 ml) and non-heparinized serum collection tubes (40 ml) in the clinical laboratory ward of the hospital. The samples were immediately transferred to the clean room under cold and clean conditions.

### NK cell expansion

Total peripheral blood mononuclear cells (PBMCs) of donors’ samples were isolated using ficol separation method based on cell density difference. Auto serum was collected after sampling (35 ml) in a Clot tube without anticoagulant (centrifuge at 2000 g–10 min).

Next, 35 ml of whole blood samples were diluted in equal ratios with phosphate-buffered sodium (PBS) [Sigma-Germany], and the final suspension was transferred gently to the pre-warmed (25 °C) ficol solution. After 20 min of centrifugation at a gradient speed of 800 g, the final mixture was separated into three layers. The middle layer was gently picked up with a pipette, transferred to another tube, and washed twice with pre-warmed (25 °C) PBS. The pellets of the cells were re-suspended in 1 ml of conditioned media containing RPMI1640 (Gibco, USA) + 10% auto serum then were counted using 4% trypan blue dye (Sigma-Germany), and simultaneously the cell viability was visually assessed. 2.5 × 10^7 cells of each donor were prepared as 5 × 10^5 cells/ml in RPMI1640 media (Gibco-USA) containing 1000 IU/ml GMP-grade recombinant human IL-2 (Milteny Biotec, USA), 10 ng/ml OKT3 (Milteny Biotec, USA), and 10% donor-serum then were seeded in T75 cell culture flasks (Nest, China). After 72 h, the conditioned media was exchanged using RPMI1640 media containing 500 IU/ml rhIL-2 and 5% auto serum. After cell seeding, media was added and flasks were incremented every three days until 21 days post-seeding; flow cytometry was used to assess the quality of the cells. Cell viability and cell count were determined using an automated cell counter (Thermofisher, USA). The total pooled cells of each donor were packaged in cell infusion bags (Milteny Biotec, USA) (containing 50 ml 9% normal saline) and labeled for different donors. Infusion bags were transferred to the Masih Daneshvari Hospital in cold condition, and finally, 2 × 10^6 cells/kg were intravenously infused into patients in the ICU ward during 20 min.

### Flow cytometry

The expanded cells were analyzed with fluorescent dye-conjugated antibody staining for NK cell purity evaluation. 1 × 10^5 cells were picked up from packed bags (ready to infusion) and stained in 100 μl PBS containing 2% fetal cow serum (FCS) [Gibco, USA] with 1ug of APC labeled anti-CD56 and FITC-labeled anti-CD3 [Biolegend, USA]. The cells were incubated in dark and cold conditions (4 °C) for 20 min. After one step of washing with PBS, the tubes containing stained cells were read in Attune NXT flow cytometry device at the BL1-BL3 channels (Thermofisher, USA).

### Clinical outcome

Clinical monitoring of patients has been performed from the first day of admission to discharge, and the main criteria were recorded every day or in some criteria mentioned below every three days. In this report, parameters are reported as follows: first hospitalization date (H), 24 (− 1, + 1), 48 (− 2, + 2) hours before and after the cell infusion date (CELL), and finally discharge or death date (D). The vital signs, laboratory variables of patients, and their physical recovery condition were monitored and obtained from the patient’s files. The clinical parameters include temperature (T), pulse rate (PR), blood pressure (BP), weight (W), and arterial oxygen saturation (O2 sat) arrhythmia were scored according to the frequent occurrence (up to 5 times in 1 h) of arterial tachycardia. Suffocation or dysphoria was recorded according to the patient’s complaint.

### Clinical laboratory tests

Routine clinical blood examinations were evaluated on patients’ samples using an automated cell counter device (Sysmex, Japan), these tests included the hematologic parameters such as complete blood count (CBC), mean corpuscular volume (MCV), hemoglobin level (Hb), and platelets (PLT) count. The hematocrit (HCT), international normalized ratio (INR), and D-Dimer level were reported from the hematology-coagulation experiment ward.

The biochemical parameters such as blood urine nitrogen (BUN) and creatinine (Cr) were measured by an autoanalyzer (Aurora Biochemistry Analyzer, USA). The quantitative C-reactive protein (CRP), aspartate aminotransferase (AST), alanine aminotransferase (ALT), alkaline phosphatase (ALP), and lactate dehydrogenase (LDH) were measured using specific kits. The serum level of interleukin-6 (IL-6) was determined by an ELISA kit. All laboratory analyses were reported from the general laboratory center of Masih Daneshvari Hospital.

### Data analyses

The efficacy of NK cell infusion in the recovery of patients was evaluated based on variations of inflammatory parameters such as ALT, AST, ALP, CRP, IL-6, D-Dimer level, lymphocyte/WBC ratio, and INR. These evaluations were performed using the SPSS software (version 25) based on the laboratory data of patients before NK cell infusion, after infusion, and at the discharge time. Due to the small sample size in both groups, the non-parametric tests were used and the significance level of variables was considered 0.05.

### Quality control of final product

The 7-day culture of the final product was carried out in vitro diagnostic (IVD) license LB-broth (Merck, USA) and Thioglycollate Broth (THIO) culture medium (Merck, USA) in aerobic and anaerobic conditions, and the final subculture was done on the seventh day in the LB-agar and Trypticase Soy Agar (TSA) culture medium (Merck, USA). Mycoplasma contamination was evaluated by the reference laboratory of Iran's genetic resources center for the purpose of product no-contamination approval.

## Results

### Characteristics of injected NK cells

**Fig. 1:**
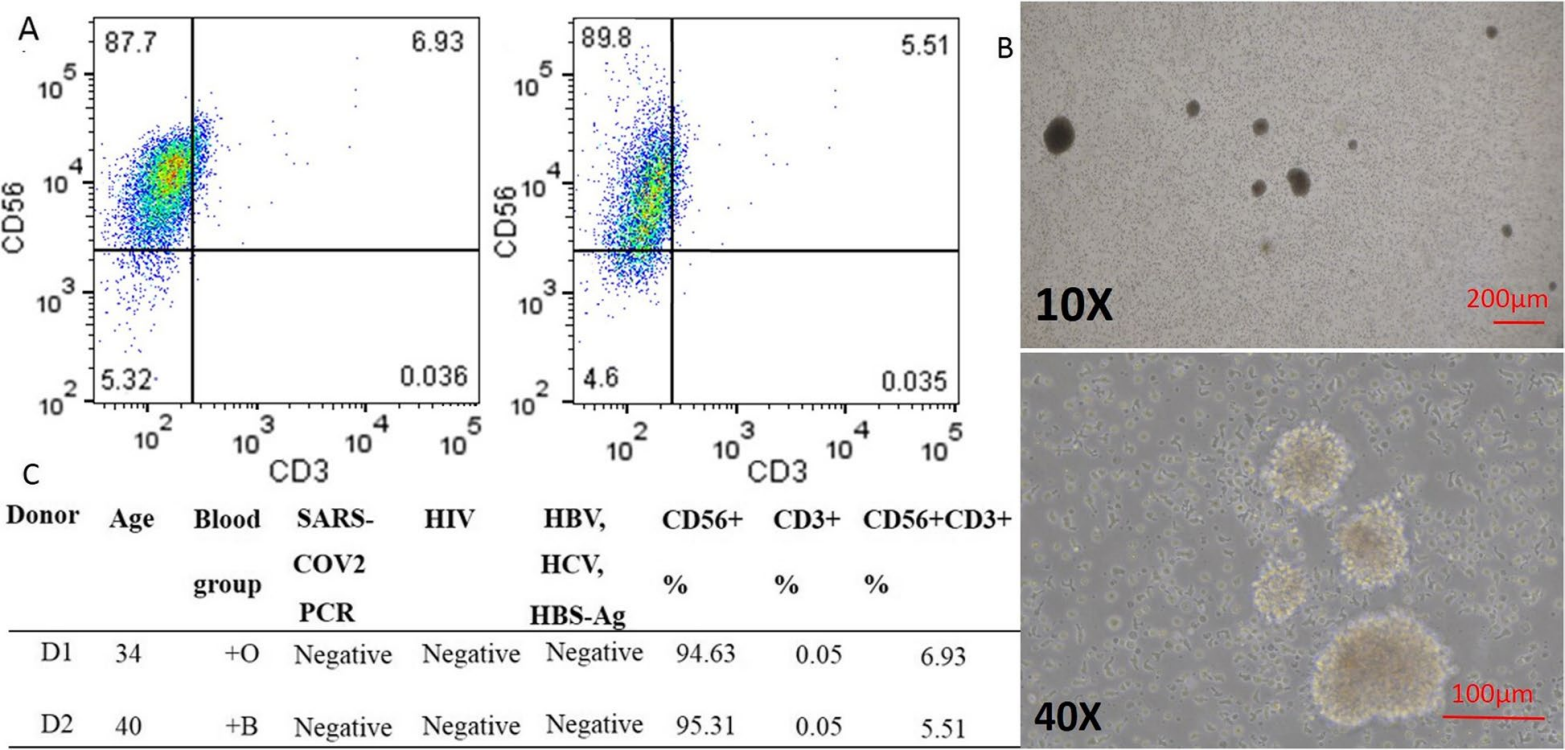
NK cell characterization. **A** The flow cytometry data for CD56 and CD3 double staining. **B** The observation of NK cells expansion in two magnifications. **C** The healthy donors’ information. General description of graphs: Generally: the red bullets are for dead patients and green tonnages are for improved patients, the circular bullets are for female patients and the square bullets are for male patients. In the control group: 1st hospitalization (**H**), the median date of the treatment period (M), and discharge or death date (**D**), in the intervention group: 1st hospitalization date (**H**), 24 (− 1, + 1), and 48 (− 2, + 2) hours before and after cell infusion (CELL)

The expanded NK cells were produced up to 6 × 10^8 cell count for both donors. The viability percentage of prepared cells was 100% in all bags before packaging. The purity of CD56 + cells in bags of D1 expanded derived NK cells was 94.63% and in D2 was 95.31% (Fig. 1A). The detailed information of expanded NK cells is listed in Fig. 1C.

### Patients characterization

This study was performed on ICU-added COVID-19 patients with acute pneumonia. All patients were classified into two groups (intervention and control) with the same characteristics in the case of gender, age, and weight. Forty percent of patients were female in the control group (2F, 3 M) and male in the intervention group (3F, 2 M). The mean age of the patients was 69.2 ± 9.25 in the intervention group and 63.6 ± 14.41 in the control group. The mean weight of the control and intervention groups is 78 ± 7.1 and 77.8 ± 14.4 kg, respectively. The duration of hospitalization in the intervention group (7.4 ± 4.5) was shorter than in the control group (10.4 ± 3.2). However, this difference was not statistically significant (*p* = 0.25, CI 95% = 8–18). One death occurred in both groups, and neither group differed from the other. The complete data are described in Table 1.

**Table 1:** The demographic information for patients

No	Code	Grouping	Gender	Age	Weight	Hospitalization	Status^a^
1	NK-C-01	Control	Female	61	76	7	D
2	NK-C-02	Control	Male	53	89	15	D
3	NK-C-03	Control	Male	66	70	10	D
4	NK-C-04	Control	Female	51	75	8	D
5	NK-C-05	Control	Male	87	80	12	E
6	NK-T-01	Intervention-D1	Male	65	65	12	D
7	NK-T-02	Intervention-D1	Female	64	85	12	D
8	NK-T-03	Intervention-D1	Male	59	99	6	D
9	NK-T-04	Intervention-D2	Female	78	65	2	D
10	NK-T-05	Intervention-D2	Female	80	75	5	E

^a^*E* expired, *D* discharged

### Safety assessment

Initially, CTCAE parameters such as fever, rash, heart attack, changes in respiratory capacity, and anaphylactic shock symptoms were closely monitored using remote patient monitoring (RPM) devices from the beginning of cell infusion until 48 h. After that, the vital signs, laboratory variables, and physical recovery progression were monitored and data were recorded according to the patient’s files According to the CTCAE checklist, no adverse effect in favor of anaphylaxis or acute reaction was seen. In some patients, mild symptoms of tachycardia (3 times arrhythmia in one hour) were reported that was negligible. Hence, the safety assessment grading was deliberated as 1 (Table 2).

**Table 2:** The safety assessment of participants

Code	Fever	Rush	Heart attack	Bronchospasm	Edema	Allergic signs	Other adverse event	Arrhythmia	Safety assessment grading
NK-T-01	None	None	None	None	None	None	None	None	+ 1
NK-T-02	None	None	None	None	None	None	None	+ 2^b^	+ 1
NK-T-03	None	None	None	None	None	None	None	+ 1^b^	+ 1
NK-T-04	None	None	None	None	None	None	Low dysphoria	+ 1	+ 1
NK-T-05	None	Low^a^	+ 2	None	None	None	None	D	+ 1

^a^The patient also had a rash on the first day of hospitalization ^b^It has been occurred 1 and 2 days after NK cell infusion

### Feasibility assessment

Project feasibility criteria were assessed prior to the initiation of the project, during the execution of the trial, and after the completion of the intervention. Our results showed acceptable feasibility in sampling and patient enrollment, access to information during the intervention, and possibility of detailed follow-up after discharge. The acceptable phrase was used for median or high grading ranges, which resulted in a feasibility assessment using a questionnaire (Table 3).

**Table 3:** The questionnaire for feasibility assessment

Sample size	Special care ward				
Daily Enter	5				
Daily Discharge/Death	3				
Total Capacity	15				
Data access	Demographic information	Hardcopy of patient file	Local access to HIS of hospital	Remote access to HIS of hospital	Full access to patients's information after discharge
Accessable options	10 +	10 +	10 +	0	5 +
Access to cause of death	No information	General information	Professional information		
Aceccable Options	0	3 +	2 +		

*HIS* Health information System

The results of the first part, which are related to the characteristics of the produced cells, are presented as follows. The results of quality control tests showed that no bacterial colony grew in the evaluated samples in the 10-day culture. These findings were confirmed by the results of the reference laboratory.

### Vital signs evaluation

**Fig. 2:**
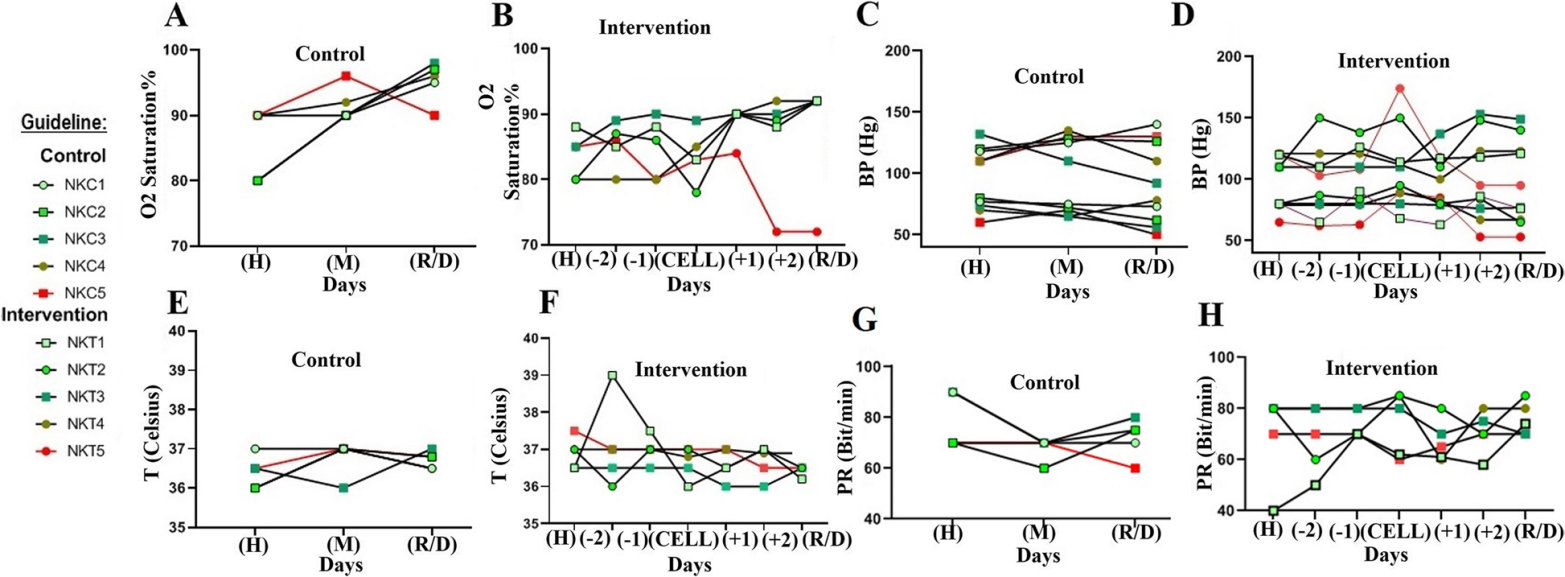
The vital signs. O2 saturation percent in all patients of the control (**A**) and intervention (**B**) groups. Blood pressure measurement in control (**C**) and intervention (**D**) groups. Body temperature of control (**E**) and intervention (**F**) groups’ patients. Pulse rate of control (**G**) and intervention (**H**) groups’ patients

A significant increase in the level of oxygen in the whole blood of all patients occurred 24 h after cell infusion, reaching 89.8% ± 2.6 (*p* = 0.03, CI 95% = 78–95) (Fig. 2A, B). On the first day of admission, individual differences in BP were statistically insignificant (*p* = 0.75, CI 95% = 9–15) in the intervention and control groups, which decreased from 138/84.2 ± 5.2 to 116.4/78.2 ± 7.9, 24 h after cell infusion. There was no statistical significance to this change (*p* = 0.33, CI 95% = 105–130) (Fig. 2C, D). The mean temperature of the intervention and control groups was approximately 36.5–37 °C on all days. In both groups, no increase in body temperature or fever was observed during hospitalization (Fig. 2E, F). The pulse rate of live patients has not changed during the hospitalization, and NK cell infusion had no critical effect on PR value (Fig. 2G, H).

### Hematologic parameters

**Fig. 3:**
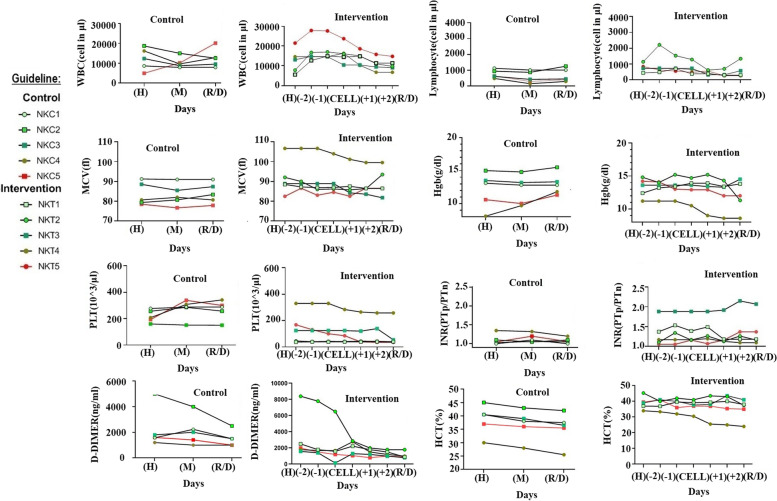
Hematologic parameters. WBC count per microliter in control (**A**) and intervention (**B**) groups. Lymphocyte count per microliter in control (**C**) and intervention (**D**) groups. The amount of MCV in control (**E**) and intervention (**F**) groups. the serumic level of hemoglobin in control (**G**) and intervention (**H**) groups. The number of platelet in control (**I**) and intervention (**J**) groups. INR ration in control (**K**) and intervention (**L**) groups. The amount of D-Dimer in control (**M**) and intervention (**N**) groups. The percentage of hematocrit in control (**O**) and intervention (**P**) groups’ patients

Total WBC and lymphocyte count were measured in all patients and were compared in both groups. The mean percentage of lymphocytes was respectively 6.6 and 7.3 in the control and intervention groups, which was not a statistically significant difference (*p* = 0.25, CI 95% = 6–8). Although the count of lymphocytes decreased immediately after the cell infusion, their count increased finally at the end of hospitalization. However, this change was not statistically significant (*p* = 0.09, CI 95% = 0.5–4) (Fig. 3A–D). The patients’ MCV level was in the normal range but one of the patients had higher MCV (NKT4). This result was in line with low hemoglobin values, which can indicate the patient's anemia. The mean MCV was 91.74 ± 9.04 in the intervention group and 83.7 ± 5.83 in the control group. The difference between MCV was not a significant difference (*p* = 0.1, CI 95% = 89–95). The MCV decreased after the injection and got closer to the normal range, but this change was not statistically significant (*p* = 0.7, CI 95% = 2–7) (Fig. 3E, F). The Hemoglobin level on the 1st day of hospitalization was 13.24 ± 1.4 in the intervention group and 12.06 ± 2.7 in the control group (not a significant difference (*p* = 0.4, CI 95% = 11.5–14)). NK cell infusion had no significant impact on the hemoglobin level in the serum (*p* = 0.48, CI 95% = 0.25–1.2). The hemoglobin level of a patient in the intervention group was much lower than the normal range, which also decreased after the injection (Fig. 3G, H).

The mean platelet count on the 1st day of hospitalization was 140 in the intervention group which decreased non-statistically significant after cell injection (*p* = 0.7, CI 95% = 110–180). The control group did not experience this problem either and never exceeded normal levels from the start of the hospitalization to discharge (Fig. 3I, J). The mean ratio of INR was 1.31 ± 0.34 in patients of the intervention group on the 1st day of hospitalization, which increased after NK cell infusion, but this change was not significant (*p* = 0.4, CI 95% = 0.12–0.36). The ratio of INR in the control group was 1.1 on the 1st day of hospitalization, which remained unchanged until discharge (Fig. 3K, L).

The normal range of D-Dimer is between 220 and 550 ng/ml in healthy people. A reduction in D-Dimer levels after NK cell infusion until discharge resulted in a significantly lower mean level at discharge as compared to the control group (*p* = 0.03, 220–350 CI 95%) (Fig. 3M, N). The mean of HCT in the intervention group and control group was almost 38.5 which had no significant difference from each other and treatments strategies had no important effect on HCT value and the results were in the normal range (35.5–44.8) (Fig. 3O, P).

### Biochemical parameters

**Fig. 4:**
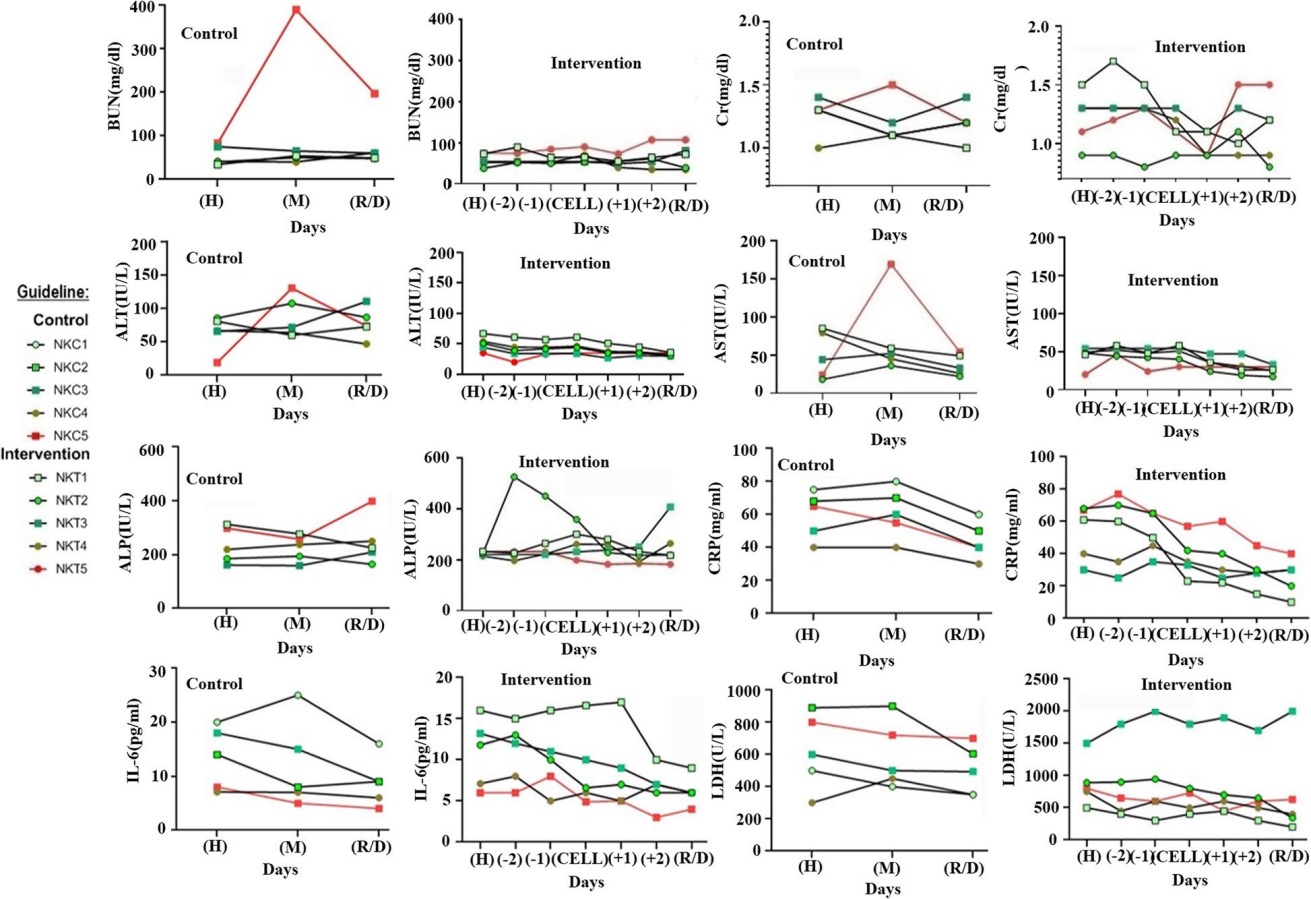
Biochemical parameters. The serumic level of BUN in control (**A**) and intervention (**B**) groups' patients. Creatinine (Cr) measurement in serum of control (**C**), and intervention groups (**D**). ALT serumic level in all patients of the control (**E**) and intervention (**F**) groups. AST serumic level measurement in control (**G**) and intervention (**H**) groups. ALP serumic level evaluation in patients of the control (**I**) and the intervention (**J**) groups. The quantitative CRP in control (**K**) and intervention (**L**) groups. The serum level of IL-6 in control (**M**) and intervention (**N**) groups. The LDH in control (**O**) and intervention (**P**) groups’ patients

The BUN level measurement daily showed that NK cell infusion had no significant effect on BUN level in the intervention group (*p* > 0.05, CI 95% = 45–65) and the amount of BUN on 1st day of admission was 54.6 ± 22 and 58.4 ± 15.3 in the control and intervention groups respectively (Fig. 4A, B). As a result of the infusion of NK cells on the first day after admission, it was found that Cr serum levels significantly decreased and reached 0.98 ± 0.1 (*p* = 0.04, CI 95% = 0.88–92). The serum level of Cr in the control group has not been changed on all days of hospitalization (Fig. 4C, D).

The normal range of ALT is 5–40 IU/lit in healthy people. The mean ALT was 63.8 ± 25.8 in the control group and 32.2 ± 4.5 in the intervention group. This difference was statistically significant (*p* = 0.03, CI 95% = 27.8–39). The amount of ALT after NK cell infusion had no significant change in the intervention group (*p* = 0.2) (Fig. 4E, F, G and H). The normal range of ALP is between 64 and 306 IU/lit in healthy patients. The serum level of ALP was 235 ± 67.5 in the control group and 224.8 ± 8.05 in the intervention group. Even though the measured ALP on all days was in the normal range, NK cell infusion decremented the serum level of ALP. However, this change was not statistically significant (*p* > 0.05, CI 95% = 200–250) (Fig. 4I, J).

The normal range of CRP and IL-6 is between 8 and 10 mg/ml and 0–16 pg/ml in healthy people, respectively. The results of patients showed that NK cell infusion could reduce the amount of CRP after infusion up to discharge date and the mean of CRP at discharge date was significantly less than the control group (*p* = 0.03, CI 95% = 2–5) (Fig. 4K, L). Similar results were observed in IL-6 (Fig. 4M, N) of the intervention group, but the average reduction was not statistically significant at the discharge date (Table 3). LDH: the normal range of LDH in healthy adults is 40 to 280 units/L. It is raised in all patients and passed the normal range. The means LDH in hospitalization date of the control group was 618 U/L and in the intervention group was 888 U/L; these values were statistically the same in both groups (*p* > 0.05, CI 95% = 600–900) (Fig. 4O, P).

### Brief evidence of efficiency

Generally, SPSS-based analysis of the efficacy-related parameters for both groups showed no significant statistical difference (*p* > 0.05) in parametric and non-parametric examinations. However, descriptive data revealed sensible differences between the groups. The discharge date and (− 1) day results of the intervention group and control group were clearly different for the inflammatory parameters LYM/WBC ratio, CRP, AST, ALT, ALP, LDH, ESR, D-Dimer, and IL-6 serum levels. To clarify the differences, we defined a difference index for each patient in the intervention groups as D cell (Table 4) which means the difference between all inflammatory parameters in “the intervention group's discharge date” and “the cell infusion date.” As well as, all results in “the control group’s discharge date” minus “the median date of hospitalization” was D-M. Consequently, these analyses showed that the D cell difference index in most patients of the intervention group was more than the D-M index in the control groups (Table 3).

**Table 4:** The serum levels of the inflammatory parameters

Difference	Parameters	LYM/WBC	CRP	AST	ALT	ALP	LDH	ESR	D-Dimer	IL-6
	Patients									
D-M	NK-C-01	− 2	− 20	− 11	− 2	− 85	− 50	0	− 733	9
	NK-C-02	26	− 20	− 15	− 6	− 50	− 296	− 27	− 1500	1
	NK-C-03	0	− 20	− 19	39	22	− 6	0	− 500	− 6
	NK-C-04	0	− 10	− 19	− 17	172	− 100	0	0	− 1
	NK-C-05	− 1.2	− 15	− 30	− 57	10	− 20	− 31	− 400	− 1
D-Cell	NK-T-01	0	− 51	− 38	− 25	− 83	− 200	− 31	1277	0
	NK-T-02	1	− 48	− 34	− 13	− 307	− 458	− 27	− 5500	− 5.8
	NK-T-03	12	0	− 21	− 4	− 190	200	− 21	− 775	− 7.2
	NK-T-04	2	− 10	0	0	− 34	− 100	− 29	− 1770	0
	NK-T-05	1.5	− 27	− 16	10	− 10	− 100	− 3	− 300	− 2

*D* = discharge, *M* = median of hospitalization, *Cell* = NK cell infusion

## Discussion

This pilot study was conducted to the evaluation of allogeneic NK cells injection into acute pneumonia patients infected by SARS-COV2. This study is the official report of the safety of NK cell injections in patients with mild respiratory distress resulting from SARS-COV2 infection. In the present study, 2 × 10^6 cells/kg were injected into each patient. The experiment was designed to confirm its safety and feasibility with low population enrolment. In this regard, no symptoms of anaphylaxis, hypersensitivity, changes in blood pressure and inflammatory enzymes, or cardiovascular complications were observed in the intervention group. To evaluate the death rate and pieces of efficacy, the control group was designed the same as the intervention group and hospitalization and death rate were reported in both groups. These achievements could suggest the infusion of ex vivo-expanded allogeneic donor-derived NK cells as a safe intervention in COVID-19 patients and its efficacy needs more data and more population enrolment. In this study, some parameters were reviewed retrospectively based on clinical files of patients whose clinical data were different from the mean and/or far from the normal range.

The safety of ex vivo-expanded allogeneic donor-derived NK cells infusion has been identified in multiple previous clinical trials for several malignant diseases [22–24]. However, this therapeutic strategy was not repetitively checked for inflammatory disease caused by viral infections. CYNK001 is an immune cell-based off-the-shelf NK cell product that is pending phase III clinical trial permission [14] and KDS-1000 as another cell-based product is prescribed for moderate COVID-19 patients in phase I/II clinical trials are some important immune cell therapies in COVID-19 treatment [25]. Harnessing the memory of NK cells would be more effective in viral infectious diseases. Seropositive donor selection for NK cell expansion might lead to more effective NK cells and we will consider this one in donor selection [11]. Although this study was performed to evaluate the safety of NK cell injection in COVID-19 patients, there is little evidence about the efficacy of this treatment on patients’ clinical outcomes. Among the most promising parameters, we achieved after the intervention was a sensible decrease in hospitalization time for patients.

Meijuan Zhen et al. have shown that antiviral lymphocytes become exhausted and inefficient in the advanced stages of the COVID-19. They have demonstrated that NK and CTL cells, which play an important role in the exposure of virus-infected cells, are decreased in number and showed an exhausted phenotype (PD-1 +). Moreover, NK cells have expressed inhibitory ligands and lost their antiviral functions [12]. Indeed, COVID-19 causes acute lymphopenia and disrupts the function of NK cells. The normal range of peripheral blood platelets in healthy people is between 150 and 450, which is increasing or decreasing in people with COVID-19 and our observation confirmed this fact and cell infusion caused to platelet decrement [26]. We expected to see a significant change in the number of lymphocytes in the patients after the cell injection; however, evidence showed that this change did not occur rapidly, and even after 24 h, there was a marked decrease in the number of lymphocytes. This may also be due to the adaptation of the immune response to the cell injection or patients’ lymphocytes carrying the virus and presenting the virus-associated NK cell activating biomarkers [27, 28]. Although an increase in the number of lymphocytes is seen in the control group, point-to-point changes were more in the intervention group. The obtained evidence showed that the mean lymphocyte count on the discharge date was higher than on the first day of hospitalization in the NK cell-receiving group, and even before the cell infusion date. Indeed, the blood urea nitrogen, creatinine, and liver enzymes are all among the parameters in this study, which did not change significantly after NK cell therapy. This fact indicates the lack of any adverse effect of applied cell therapy on the function of internal organs. Some out-of-mean differences in the intervention group can be addressed, For example, a patient numbered NKT3 had a high INR value who had a history of using a cardiac stand and long-term anticoagulant drugs. Patient numbered NKT4 was suspected of having megaloblastic anemia in terms of clinical symptoms and Alzheimer's drug usage history so her low hemoglobin level and high MCV mount from the first admission date to the discharge date would be justified due to her history. The patient numbered NKT5 had an underlying neurological disease and had received some drugs causing primary leukocytosis, which resolved after hospitalization, and her WBC count decreased. The blood O2 saturation increment in the intervention group was a piece of pieces of evidence in favor of NK cell infusion efficacy. It is very promising data, but these results are not statistically significant compared to the control group. It will be more reliable in the phase II study with more patient evaluation. However, allogeneic NK cell infusion in viral infectious disease is one of the newest topics in immune cell therapy for infectious diseases, but any data on this issue will help to increase the clinical knowledge of NK cell therapy in inflammatory disorders caused by viral infections [9]. Due to the small number of patients enrolled in this study, we could not compare the effect of cells obtained from the SARS-COV-2 seronegative healthy donor with the healthy seropositive donor. Nevertheless, this idea would be accurately evaluated if a second injection could be given. The slight evidence of NK effectiveness in the intervention group was very promising and the descriptive results are completely in favor of NK cell infusion. These data are also confirmed by the study results conducted by Lara Herrera et al. They have revealed pieces of evidence of chimerism and proliferation of memory NK cells [13]. NK cell dysfunction has been proved in COVID-19 patients, the current study was executed to compensate for this problem in patients with acute infectious, our results showed that NK cell infusion is safe in viral infection and it can help to improve the clinical manifestations in patients with respiratory involvement [8].

The number of injected cells has been in the range of studies similar to cellular immunotherapy of viral infections such as COVID-19, In a similar clinical trial (code: NCT04280224) in China, the number of infused NK cells was reported between 1–10 × 10^6 cells/Kg (twice a week) [29]. Moreover, phase I and II clinical trials for allogeneic injection of NK cells (NCT04365101) are underway to evaluate the CYNK-001 cellular drug (injection of a single dose of allogeneic NK cell drug from a healthy donor). The NK cells of that study were taken from the placenta and fetal umbilical cord blood. They would be injected three times (days 1, 4, and 7) with different doses in 86 patients suffering from COVID-19 pneumonia [4]. The US Food and Drug Administration (FDA) is investigating the CYNK-001 cellular drug for COVID-19 treatment. This study is currently in phase II clinical trial. This cell product has been injected into eight patients, of which six have been successfully treated without any adverse effects, and the disease state has returned from its severe stage [30]. As far as, GC LabCell as a biotechnology company in Korea uses a combination of monoclonal antibodies and NK cells to treat COVID-19 infection in cancer patients [15].

Our clinical study had some limitations, e.g., the trial lacked randomization and comparison with a small sample size, which made it difficult to assess the efficacy of allogeneic NK cell therapy. This was a pilot study and we did not aimed to have a high power for hypothesis testing. On the other hand, safety of patients was dominated based on ethics committee clearance. The committee does not grant the permission to enroll a higher number of participants, due to our main purpose for safety analyses. Thus, we proposed to enroll 16 patients (8 patients in intervention and 8 patients in control group) as minimum number required for this phase. Finally we managed to enroll 10 patients (5 in each arm) to conduct the study. We aimed to conduct the following phase II study for efficacy. It could be concluded that our trial demonstrates the safety of donor-derived allogeneic natural killer cells to be administered in patients with lower to moderate respiratory disease. The allogeneic natural killer cells with suitable doubling times can be expanded efficiently in the cleanroom and it is a good result of the feasibility of GMP-grade NK cell production to execute future clinical trials. This study might be introduced as a case-series evaluation that observes the safety of NK cell injections in COVID-19 patients. Although there are no critical adverse effects in none of the patients, for results that are more conclusive, the trial should be continued in the next phase. The project execution was feasible in terms of patient enrollment and evaluation of safety evidences, but further studies and higher number of patients is required to confirm the effectiveness of the treatment.

### Key recommendations

- The patients with renal complications would be added in exclusion criteria
- The patient enrolment in treatment group should be normalized with control group in case age and sex
- The baseline of blood sugar should be checked and diabetic patients should be withdrawn from study
- The history of asthmatic patients should be checked
- The CD4/CD8 ration should be checked before/after cell therapy
- The raw data of patients should be read on line and decision making would be checked by specialist

### Supplementary Information

**Additional file 1: Supplementary Table S1.** AGREE Reporting Checklist 2016.

## Data Availability

The data generated during the study to support the findings are available upon request from the corresponding author (Dr. Ramin Sarrami Forooshani) upon reasonable request.

## Abbreviations

NK: Natural killer
COVID-19: Coronavirus Disease 2019
SARS-COV2: Severe acute respiratory syndrome coronavirus 2
LIC: Low inspiratory capacity
CTCAE: Common Terminology Criteria for Adverse Events
GCP: Good clinical practice
CMV: Cytomegalovirus
TRAIL: Tumor necrosis factor-induced apoptosis ligand
EMCV: Encephalomyocarditis virus
IFN-α, IFN-γ: Interferon-alpha and gamma
CTL: Cytotoxic T lymphocytes
RT-PCR: Reverse-transcription polymerase chain reaction
VARID: Viral-associated respiratory infectious disease
PBMCs: Peripheral blood mononuclear cells
LIC/TLC: Low inspiratory capacity/total lung capacity
PBS: Phosphate-buffered sodium
GMP: Good Manufacturing Procedure
T: Temperature
PR: Pulse rate
BP: Blood pressure
W: Weight
CBC: Complete blood count
MCV: Mean corpuscular volume
Hb: Hemoglobin level
PLT: Platelets
HCT: Hematocrit
INR: International normalized ratio
IL-6: Interleukin-6
CRP: C-reactive protein
AST: Aspartate aminotransferase
ALT: Alanine aminotransferase
ALP: Alkaline phosphatase
LDH: Lactate dehydrogenase
BUN: Blood urea nitrogen
